# Comparing the spatio-temporal organization of joint spiking and local field potential oscillations in motor cortex

**DOI:** 10.1186/1471-2202-13-S1-P127

**Published:** 2012-07-16

**Authors:** Michael Denker, Lyuba Zehl, Thomas Brochier, Alexa Riehle, Sonja Grün

**Affiliations:** 1 Institute of Neuroscience and Medicine (INM-6), Forschungszentrum Jülich, Germany; 2 Institut de Neurosciences de la Timone (INT), UMR 7289, CNRS - Aix Marseille Univ., Marseille, France; 3 Theoretical Systems Neurobiology, RWTH Aachen University, Germany; 4 RIKEN Brain Science Institute, Wako-shi, Japan

## 

Oscillations of the local field potential (LFP) are regarded as a signature of synchronized activity in neuronal networks. In primary motor (MI) and premotor (PM) cortex, LFPs typically exhibit such oscillatory activity in the beta range (15–30Hz) during an instructed delay [1]. These oscillations tend to display a wave-like propagation across the cortical surface [2]. In parallel, temporally precise, behavior-related spike synchronization is often observed during periods of movement preparation and expectation [3]. In a previous study we demonstrated that the occurrence of significant spike coincidences is dependent on the phase of LFP beta oscillations [4]. In order to extend these studies to include positional information, we here study how the spatio-temporal organization of the LFP activity across cortical distances of several millimeters is related to that of spike synchronization [5].

Two monkeys were trained to press a switch with one hand, and then to grasp and pull an object using either a *Side Grip* or a *Precision Grip*. The force on the object could be either low or high. In order to allow the monkey to prepare the movement, the grip type was revealed at the beginning of an instructed delay of 1 s before the GO signal. In contrast, the force information was encoded by the GO signal itself. LFP and single unit activity was recorded simultaneously from a 100 electrode Utah array implanted at the MI/PMd border.

We analyze oscillatory activity in the beta band with respect to grip type and cortical position. Based on the phase synchrony of LFPs across electrodes, we quantify the spatial inhomogeneity of LFP propagation by its direction and speed in a time-resolved manner. In parallel, we compute spike correlations that significantly exceed chance level [5] as a function of temporal, spatial, and directional parameters. We find that the likelihood of synchronized spiking is behaviorally modulated in time, and decreases with distance between the two recorded neurons. Finally, we compare the spatial distribution of spike synchrony (represented as a graph of neurons exhibiting significant spike coincidences), and synchrony expressed by LFP oscillations in different epochs of the experimental paradigm.
